# Assessing emotions conveyed and elicited by patient narratives and their impact on intention to participate in colorectal cancer screening: A psychophysiological investigation

**DOI:** 10.1371/journal.pone.0199882

**Published:** 2018-06-28

**Authors:** Teresa Gavaruzzi, Michela Sarlo, Francesca Giandomenico, Rino Rumiati, Francesca Polato, Franca De Lazzari, Lorella Lotto

**Affiliations:** 1 Department of Developmental Psychology and Socialization, University of Padova, Padova, Italy; 2 Department of General Psychology, University of Padova, Padova, Italy; 3 Padova Neuroscience Center, University of Padova, Padova, Italy; 4 Gastroenterology Unit, S. Antonio Hospital, Padova, Italy; University of Auckland, NEW ZEALAND

## Abstract

In the context of colorectal cancer screening, we aimed to compare the effectiveness of different emotion-laden narratives, to investigate the specific emotions elicited at both subjective and physiological levels, and to test the effects of emotions explicitly expressed by the narrative character. Study 1 used a between-participants design comparing four conditions: relief-based narrative, regret-based narrative, control (test-uptake only) narrative, and standard invitation material (no-narrative condition). Study 2 used a mixed design, with the narrative content as a within-participants factor and whether emotions were expressed by the narrative character or not as between-participants factor. The main outcome measures were: intention to undergo testing (Studies 1 and 2), knowledge, risk perception, proportion of informed choices (Study 1), subjective emotional responses, changes in skin conductance, heart rate, and corrugator muscle activity (Study 2). In Study 1, relative to the non-narrative condition (51%), only the relief-based narrative significantly increased intention to undergo testing (86%). Relative to the standard invitation material, the narrative conditions did not decrease knowledge, alter risk perception, or decrease the proportion of informed choices. In Study 2, the relief-based narrative elicited the lowest self-reported negative affect, and received greater implicit attention, as suggested by the larger heart rate decrease. Making the emotions experienced by the narrative character explicit decreased negative affect, as indicated by the lower skin conductance and corrugator responses during reading. Our findings provide support for the use of a relief-based narrative with emotions expressed by the character in addition to the standard information material to promote colorectal cancer screening.

## Introduction

Cancer screening tests allow for detection of cancer at an early stage, when the benefits of treatment are higher and when more conservative treatments are usually possible [1]. Specifically, colorectal cancer (CRC) screening has been shown to reduce CRC incidence and mortality in randomised trials [2–5]. Testing modalities for CRC screening include stool-based tests, such as the faecal occult blood testing (FOBT) and the faecal immunochemical test (FIT), and techniques to see inside the colon directly, usually through endoscopy (colonoscopy or flexible sigmoidoscopy). Stool-based tests are based on the fact that small amounts of blood in the faeces (not visible to our eye) might be a sign of polyps or cancer in the colon. These tests are less invasive than endoscopy, do not require preparing the bowel and dieting before the exam and can be performed at home, using a kit to collect samples to be sent for analyses. If occult blood is found, further exams (usually colonoscopy) are needed to ascertain the cause of the bleeding.

At the European level, population-based screening tests aimed at finding early signs of CRC have been recommended since 2003 [6], and guidelines to ensure the high quality of CRC screening have also been promoted [7]. An essential element for the success of mass screening programmes is the rate of uptake of the test. While there are large geographical variations in the implementation of screening programmes in Europe, low uptake rates are an issue of concern [8]. In Italy, the screening programme usually entails FIT every two years from 50 to 69 years of age. The most recent estimate of uptake is 47.1% [9], which is slightly above the threshold for acceptability (45%), but far from the recommended rate threshold of 65% [10].

Several methods to increase participation in organised screening programmes have been considered [11–12]. While many methods are based on interventions that target the organisation level, such as having the general practitioner sign the invitation letter, using phone call reminders, or sending pre-notification letters, there are other interventions more deeply grounded on the psychological literature that could be used to motivate screening uptake. In particular, the use of *narratives* has been suggested as a way to promote informed uptake of cancer screening [13–16].

Narratives (also referred to as testimonials, anecdotes, exemplars or patient stories) are stories about other people who had a similar experience or faced a similar decision [17]. They are complex interventions that vary widely in their purpose, content, form, and context [18–19]. The circumstances under which narratives are effective and the mechanisms underlying their persuasive influence remain unclear, with experts calling for more extensive research [17,19].

Mixed evidence on the effectiveness of narratives has been found in the literature, also in the context of CRC screening, with some supporting their effectiveness and others not or only in certain circumstances. For example, although narratives were found to increase intentions to undergo screening [20], they were not effective in increasing the actual uptake of the test when used in addition to pre-invitation letters [21]. The differences between the studies need to be considered. For example, while the previous studies by McGregor and colleagues [20–21] were conducted in the UK, where a public screening programme is available, most of the other studies conducted on this topic were based in the USA [22–29] where cancer screening is typically volitional, health insurance coverage and cost are issues to be considered, and participants usually have also more choice in terms of which screening test to undergo (e.g., sigmoidoscopy, colonoscopy, or FIT).

Another related difference between studies on this topic concerns the manipulation of the focus of the narratives. While some studies tailored the narrative to target some participants’ characteristics or factors known to predict uptake (e.g., barriers to colonoscopy [23]; living arrangements [30]), this is not suitable for the context of mass screening invitations, where age is the only criterion used to send invitations.

Finally, another important difference that is worth mentioning pertains to the content type of the narratives, varying from people who failed to participate or delayed screening and were later found to have cancer [28], to people who only discuss their decision making about testing [22–23]. It is therefore possible that differences in narrative perspectives, in addition to programme format and content, contribute to the mixed results found on the effectiveness of narratives in promoting CRC screening.

While the study of narratives in CRC screening needs further refinement, there is general agreement that emotions play a central role in determining the effectiveness of narratives. In contrast with standard health educational material, where information is presented in a didactic style, often providing epidemiological data, list of risk factors and recommended actions, narratives—by representing the typical way that people use to communicate with each other—convey essential social-affective information, producing cognitive and emotional effects that might positively affect attitudes, intentions, and behaviour change [13,15]. Although researchers acknowledge the central role played by recipients’ emotional responses in narrative persuasion [31], few studies have measured the emotions elicited during the processing of the narrative text, yielding inconclusive results. For example, both positive (happiness) and negative emotions (anger, sadness, disgust, happiness, surprise, and fear) to a cancer-related storyline were found to predict information seeking and to increase behaviour change [32]. In contrast, when using a story about a person dealing with the consequences of cancer it has been shown that (among fear, sadness, surprise, and compassion) only fear significantly predicted the intention to perform a self-exam [33].

Further research is needed to better understand which emotions are elicited by a given narrative and to clarify their impact on recipients’ information processing and motivation to engage in active behaviour. Indeed, negative emotions might both increase information processing [34], and evoke defensive responses [35], which in turn might affect attitudes and intentions even in the absence of conscious emotional experience [36]. Indeed, emotion-related nonconscious somatic signals are able to strongly affect decision-making and to guide advantageous behaviour [37].

The present research aimed to assess the explicit and implicit emotional responses to different kind of narratives and their impact on people’s intentions in the context of promoting informed uptake of colorectal cancer screening. Physiological measures have been used to ‘complement and clarify insights gleaned from ratings of emotions that people are willing and able to report’ (p181)[38]. Specifically, the corrugator electromyographic (EMG) activity has been shown to be a reliable implicit index of emotional valence, as response amplitude is positively correlated with experienced unpleasantness [39]. The concurrent recording of heart rate and skin conductance allowed the detection of a response pattern indexing a defensive motivational set (skin conductance increase associated with cardiac acceleration) *vs*. an attentional set (skin conductance increase associated with cardiac deceleration) [40]. By investigating the relationship between emotional reactivity (both subjective and physiological) to narratives and intention to undergo FIT screening, we could explore the role played by emotional reactions in modulating narrative effectiveness. To the best of our knowledge, to date there are no studies in the health context assessing the physiological changes associated with the reading of narratives.

In the context of standard screening invitation, people typically think about the possible outcomes and mentally simulate how they would feel about them. As a result, people may experience anticipated regret by thinking about the case in which an adverse outcome would happen. In cancer-related decisions, anticipated regret plays an important role [41] and experiencing high levels of anticipated regret strengthens people’s intentions to engage in the action [42]. Additionally, anticipated regret increased screening uptake in the context of cervical cancer [43], while in the context of FOBT promotion its effect was found to be limited to people with low intentions [44] and it did not increase intention to screen when associated with a negative-framed message [45].

In the context of cancer detection, relief has also been suggested to be key to the persuasiveness of the message [31]. The relief and peace of mind associated with being reassured have been shown to be highly valued by patients in other screening contexts (e.g., breast cancer [46] and prostate cancer [47]). Moreover, feeling reassured and relieved have been shown to increase repeated adherence to screening testing by reducing anxiety in the context of breast cancer screening [48], and to be associated with higher likelihood of participation in the context of prostate cancer screening [47]. In the context of colorectal cancer screening, relief and peace of mind have received less attention, although they emerged as central theme in qualitative research about the benefits that people find in CRC screening [49]. Some evidence suggests that the relief experienced imagining a negative FOBT test result increases the intention to undergo the screen, when associated with a positive-framed message [45]. In summary, both anticipated regret and relief are good candidates as emotions that could motivate CRC screening when described in a narrative, but they have not been previously investigated in the same study, nor by using narratives.

Besides the role played by specific emotional contents in narrative effectiveness, another issue largely neglected in the relevant literature regards the way such emotions are implemented in the narrative scenario, as they can be explicitly expressed by the narrative character or not. To the best of our knowledge, this issue has never been explored before. Indeed, a recent comprehensive review [19] suggests that the expression of emotions in the narrative text is a promising characteristic associated with increased persuasiveness of the narratives. But a closer look at the studies considered by the authors shows that what is considered as high vs. low emotional content varies greatly, e.g. from imagined vs. real outcomes [50] to physical vs. psychological consequences [51]. Moreover, high vs. low emotional content are also characterised by different formats, including emotional adjectives, diverse punctuation, and emoticons [52].

The aim of Study 1 was to compare the effectiveness of different emotion-laden narratives in promoting intention to adhere to screening invitation. Specifically, we compared the standard invitation material (no-narrative condition) with three narrative conditions: relief-based (negative test result), anticipated regret-based (positive test result, early stage cancer treated successfully), and control (test-uptake only: the character had the test and is waiting for result) narratives. The aim of Study 2 was twofold: a) to investigate the specific emotions elicited by the relief-based, anticipated regret-based, and control narratives at both subjective and physiological levels, and b) to test the effects of emotions explicitly expressed by the narrative character by comparing two versions of the emotion-laden narratives, one in which the character explicitly reported her emotions at the end of the narrative and one in which emotions were not reported.

## Study 1

### Materials and methods

#### Participants

The study received approval from the Psychology Ethics Committee of the University of Padova (protocol number: 1188). Participants were recruited through flyers in community gathering places not related to health in three provinces (Padova, Verona, and Vicenza) of the Veneto Region. Participants had to be 45 to 65 years of age. Having been or being currently diagnosed with colorectal cancer was the only exclusion criteria. Data were collected between May and July 2012. One hundred forty-five Italian participants (75 females) consented to participate and completed the study.

#### Materials

Participants received an envelope to simulate the invitation from the local CRC screening centre. The envelope contained an invitation letter and an information leaflet (S1 Appendix), both adapted from those used by the local cancer screening programme. Accordingly, the invitation letter provided basic information and set a day for invitees to collect the FIT kit at their local health department. The information leaflet provided more detailed information about CRC and screening. In the no-narrative condition, no additional material was provided. In the other three conditions, the envelope included an additional sheet with one of the three narratives. Each narrative was introduced as the experience from someone who participated in the screening programme. A vignette presented the text of the narrative (S2 Appendix).

The control narrative described the experience of someone who had the test and was waiting for the result. The relief-based narrative described the experience of someone who had a negative test result; the emotional content of relief and peace of mind was conveyed in the narrative by the sentence that reads “Now I know I’m ok, and I don’t have to think about it for at least another two years”. The (anticipated) regret-based narrative described the experience of someone who had a positive test result, with the follow-up testing revealing an early stage cancer that was removed successfully; the emotional content of anticipated regret was conveyed in the narrative by the sentence that reads “I don’t want to think about how it would have ended if I didn’t take the screening test”.

The text was in first person and gender neutral, i.e., it was not possible to infer the gender of the character. The narratives were developed based on the literature on narratives and on the literature on the use of anticipated-regret and relief information in CRC screening, in conjunction with CRC clinical experts (FP, FDL). The three narratives were equivalent in length and readability, measured both with the Flesch-Vacca index [53], which is the adaptation of the Flesh index for Italian, and the Gulpease index [54], which is a readability index developed specifically for Italian. Moreover, in a pilot study we established that the three narratives did not differ on other dimensions (i.e., credibility, vividness, and the degree of involvement and identification with the narrative) previously found to affect the persuasiveness of narratives. The judgments expressed by 45 participants showed that these dimensions did not differ between the three narratives (*p* > .189). Similarly to the invitation letter and the information leaflet, the narratives were printed on one sheet of A4 paper, which was folded in three parts in order to fit in the envelope.

#### Procedure

Participants who agreed to participate were given an appointment at their home, where they signed the consent form and received the information pack that the researcher would later collect. Each participant was assigned to one condition following a Latin square, after having stratified for sex and age group (45–54 and 55–65 years). Participants were first instructed to imagine having received an envelope from their local cancer screening centre including the following material: invitation letter, information leaflet, and narrative.

After having read the material at their own pace, participants completed a paper-based questionnaire. First, they rated their intention to undergo screening by answering the question: “If you were to decide now, would you accept the invitation received?”, using four answer options: 1) certainly yes, 2) probably yes, 3) probably no, and 4) certainly no.

Knowledge, risk perception, and a measure of informed choice were assessed afterwards in order to ascertain that these variables were comparable in the four conditions (i.e., the no-narrative condition and the three narrative conditions). *Knowledge* of the information provided in the leaflet was assessed with 10 true/false questions (e.g., “In order to have the FIT test, you have to go to the hospital” or “Before the test you can eat normally. It is not necessary to avoid specific foods, such as read meat”; range: 0–10).

*Risk perception* was assessed with two questions, following the distinction between cognitive and affective components of cancer risk perceptions [55]. The *Perceived cognitive likelihood* question, emphasized logic, rationality, accuracy, and objectivity before asking “If I don’t do the test, I think that the likelihood of having late stage colorectal cancer some time during my lifetime would be….” (on a 5-point scale from 1 = “extremely low” to 5 = “extremely high”). The *Perceived affective likelihood* question focused participants on intuitive gut-level responses and feelings of risk before asking “If I don’t do the test, I feel that the likelihood of having late stage colorectal cancer some time during my lifetime would be….”.

Similarly to previous studies [56–57], the *Informed choice* measure was based on Marteau’s multidimensional measure of informed choice [58] and was defined as adequate knowledge combined with intention to undergo testing congruent with attitudes toward screening. Attitudes were assessed through the degree of agreement (on a 4-point scale, from 0 = not at all, to 3 = extremely) with 9 items (e.g., “Having the test would make me feel I am doing something positive for my health” and “If I had something, I would rather not know it”, reverse coded). The attitude score was computed as the sum of the answers (range: 9–36). Choices were categorised as informed if knowledge was adequate (defined as a score ≥ 8 on a 0–10 scale), and the intention to undergo testing (yes for “certainly yes” or “probably yes” and no for “certainly no” or “probably no”) was congruent with attitudes (positive attitude if score was ≥ 27, negative if score was < 27, on a scale from 9 to 36).

Additionally, we collected information on factors that could affect intention to undergo screening, namely: age, sex, occupation, education, previous CRC screening test(s), other cancer screening test(s), family members or friends with CRC cancer. After having completed the study, participants were thanked and debriefed.

#### Data analysis

Sample characteristics were summarised with means and standard deviations or frequencies and percentages. Considering the low frequency of responses “probably yes”, “probably no” and “certainly no”, these answers were grouped together, and *Intention to undergo FIT* was analysed using a binary logistic model (“certainly yes” 1, other answers 0), with the condition (no-narrative condition, control narrative, regret-based narrative, relief-based narrative) as predictor. A second model included also the following predictors: previous CRC screening tests (yes 1, no 0), other cancer screening tests (yes 1, no 0), family members and/or friends with CRC cancer (yes 1, no 0), age, and sex (male 1, female 0). For both models, the analyses were repeated with the different conditions as reference categories, in order to allow the estimate of odd ratios for all the comparisons between conditions.

Knowledge and risk perception measures were analysed using analyses of variance (ANOVAs), with *Condition* (no-narrative condition, control narrative, regret-based narrative, relief-based narrative) as between-participants factor. The proportion of informed choices (informed 1, not informed 0) was analysed with a binary logistic regression model with the condition as predictor.

For all logistic regressions, effect sizes are reported using odds ratios.

### Results

The socio-demographic characteristics of the sample are reported in Table 1. Baseline randomization checks were conducted and showed no differences (S3 Appendix).

**Table 1:** Socio-demographic characteristics of participants.

	Study 1	Study 2
**Number of participants**	145	60
**Age**		
Range	45–65	44–50
Mean (SD)	54.32 (5.37)	47.68
Mdn	55	48
**Sex**		
Females	75 (51.7%)	32 (53.3%)
Males	70 (48.3%)	28 (46.7%)
**Education**		
Middle school	42 (29.0%)	4 (6.7%)
Vocational school	24 (16.6%)	5 (8.3%)
High school	52 (35.9%)	27 (45.0%)
University degree	13 (9.0%)	18 (30.0%)
Other	14 (9.7%)	6 (10.0%)
**Occupation**		
Office workers/ Employees	60 (41.4%)	38 (63.3%)
Professionals	24 (16.6%)	13 (21.7%)
Retired	23 (15.9%)	0 (0.0%)
Housewives	22 (15.2%)	1 (1.7%)
Other	15 (10.3%)	8 (13.3%)
**Previous CRC screening test(s)**		
Yes	69 (47.6%)	9 (15.0%)
No	71 (49.0%)	51 (85.0%)
**Other screening test(s)**		
Yes	99 (68.3%)	44 (73.3%)
No	46 (31.7%)	16 (26.7%)
**Family or (close) friends with CRC**^**a**^		
Yes	93 (64.1%)	22 (36.7%)
No	52 (35.9%)	38 (63.3%)

^a^ in Study 1 family or friends, in Study 2 family or close friends.

**Fig 1:**
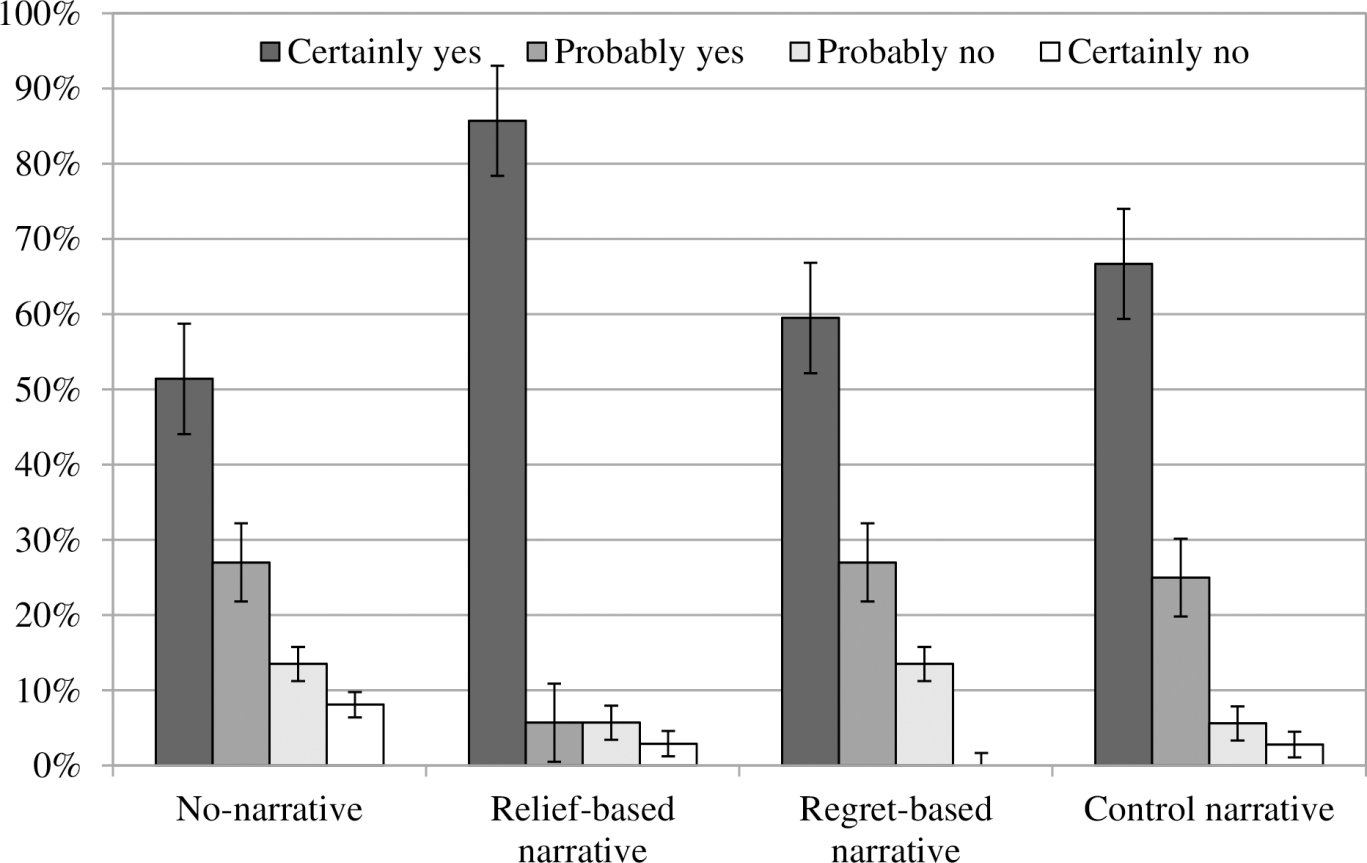
Intention. Intention to undergo screening as a function of the condition (no-narrative condition, control narrative, regret-based narrative, relief-based narrative).

As shown in Fig 1, participants were significantly more likely to indicate that they would definitely undergo FIT when reading the relief-based narrative (85.7%) than the no-narrative condition (51.4%, OR = 5.684, *p* = .003). The relief-based narrative was also more effective than the regret-based condition (59.5%, OR = 4.091, *p* = .017), and slightly more effective than the control narrative, although this difference was not statistically significant (66.7%, OR = 3.000, *p* = .066). The regret-based condition and the control condition did not differ from each other (*p* = .524) or from the no-narrative condition (*p* = .483 and *p* = .186, respectively).

When controlling for age, sex, previous CRC screening tests, other cancer screening tests, relatives and significant others with bowel cancer, the pattern of results was very similar (relief-based narrative vs. no-narrative condition OR = 4.230, *p* = .021; vs. regret-based narrative OR = 3.493, *p* = .045; vs. control narrative OR = 2.601, *p* = .134; other comparisons *p* > .355). Only having undergone previous CRC screening tests was associated with higher intention to undergo screening (prior test(s) vs. no OR = 3.130, *p* = .013; other variables *p* > .236).

The ANOVAs and the binary logistic regression showed that the four conditions did not differ for knowledge (*M* = 7.21, *SD* = 1.92, *p* = .686), risk perception measures (Cognitive: *M* = 3.36, *SD* = 1.10, *p* = .578; Affective: *M* = 3.26, *SD* = 1.04, *p* = .673), attitudes (*M* = 29.25, *SD* = 4.87, *p* = .410), and proportion of informed choices (42.4%, p = .828).

### Discussion

Our findings are in line with previous research supporting the promising nature of narratives in promoting CRC screening [20,25,59]. Moreover, they also suggest that not all narratives are alike, and that FIT testing may be better promoted by relief-based narratives, regardless of previous CRC screening behaviour. While the existing evidence suggested anticipated regret as a potential candidate for CRC screening promotion, the intention to undergo testing with the regret-based narrative was comparable to that of the no-narrative condition.

It has been hypothesised that narratives may reduce attention to or may distract resources from factual information [60–61], but our data suggest that narratives, when supplementing currently used information material, do not decrease knowledge, do not alter risk perception and do not reduce the proportion of informed choices.

A limitation of Study 1 is that emotional contexts were described through the last sentence of the narratives, but emotions were not expressed directly by the narrative character nor were measured in the participants. Therefore, we did not assess whether the narratives effectively conveyed anticipated regret and relief or whether they evoked any emotion in the participants. Moreover, previous literature suggests that cancer-related messages are likely to elicit more than one emotion, some of which may enhance persuasion and some of which may have the potential to work against persuasive goals, especially if the provoked emotions differ from those intended by the writer of health material [31]. Therefore, it is crucial to measure both the conscious and unconscious emotional reactions of participants. We addressed this issue in Study 2, by investigating the emotions elicited by the narratives at both subjective and physiological level. Furthermore, Study 2 aimed to investigate whether narratives, with or without emotions, explicitly expressed by the main character differentially modulate emotional responses and intention to undergo screening.

Participation was limited to people who were close to the screening age but had not yet been invited by the screening programme, in order to minimise previous exposure to similar informative material and to minimise the effect of previous decision-making about CRC screening.

## Study 2

### Materials and methods

#### Participants

The study received approval from the Psychology Ethics Committee of the University of Padova (protocol number: 1188) and informed consent was obtained from all individual participants included in the study. Sixty Italian participants (32 females), aged 44 to 50 years, were recruited through flyers in Padova, and appointments were scheduled by phone. Having been or being currently diagnosed with colorectal cancer was the only exclusion criteria. Participants were randomly assigned to one of two narrative emotion groups, narratives with expressed emotions (EE, n = 32, 17 females) or narratives with no expressed emotions (NEE, n = 28, 15 females). Participants were paid €15 for their participation. Data were collected between October 2012 and December 2013.

#### Stimulus material

The stimulus material was adapted from Study 1 and was presented as text through a series of 12 computer screens (4 for the invitation letter and 8 for the information leaflet). Similarly to Study 1, three types of narratives were used: control narrative (test-uptake only, waiting for test results), relief-based narrative (negative test result), and (anticipated) regret-based narrative (positive test result, early stage cancer, removed successfully). NEE narratives described the events of the story but did not include a description of the emotions felt by the narrative character, whereas this additional paragraph was added at the end of the story in EE narratives (see S2 Appendix).

#### Physiological recording

Electrocardiogram (ECG) was recorded using Ag/AgCl surface electrodes placed on the subject’s chest in a modified Lead II configuration. The ECG signal was recorded with a 0.5–500 Hz bandpass filter. Corrugator supercilii muscle activity was measured using miniature Ag/AgCl electrodes attached on the left side of the face over the corrugator supercilii muscle regions. The raw electromyogram (EMG) signal was recorded with a 50–500 Hz bandpass filter. All impedances were kept below 10 KΩ.

Skin conductance level (SCL) was recorded with Ag/AgCl electrodes attached to the palmar surface of the middle phalanges of the first and second fingers of the non-dominant hand. A GSR module (Brain Products, Germany), provided a 0.5 V constant voltage across electrodes. The SCL signal was recorded in DC, with a low-pass filter set at 20 Hz.

ECG, EMG and SCL were amplified and filtered on a V-Amp amplifier (Brain Products, Germany), digitized at 1000 Hz (24 bit AD converter, accuracy 0.05 uV/LSB) and stored on to a Pentium IV computer. Data acquisition was implemented by BrainVision recorder 2.0 software (Brain Products, Germany).

#### Procedure

Upon arrival at the University laboratory, participants were given information about the study, and their written informed consent was obtained. Participants were then seated in a comfortable chair in a dimly lit room and physiological sensors were attached. They then rested for 10 min, to allow adjustment to the experimental setting.

Afterwards, participants filled the paper-based version of the Italian version of the State-Trait Anxiety Inventory questionnaire (STAI-Y1 [62]) to assess state of anxiety. On the computer screen, participants read general information on CRC screening. To allow different reading speed, they were instructed to press the space bar to proceed throughout the screens. After having read the general information, participants rated their baseline intention to undergo testing on a Likert scale ranging from 1 (“Definitely no”) to 9 (“Definitely yes”). Then, a slide instructed participants that they would be presented with three stories from other people who underwent screening. After a 30-s baseline period, during which a slide instructed participants to remain relaxed and still, the three narratives were presented in random order across participants. After each narrative, participants rated the intensity of fear, anger, sadness, disgust, surprise, joy, and anxiety elicited by each story on a Likert scale ranging from 1 (“not at all”) to 9 (“extremely”), and their intention to undergo testing. The stimulus material was presented using the E-prime software package (Psychological Software Tools, Inc.).

At the end of the experimental session, participants completed the paper-based version of the Italian versions of the STAI-Y2 [62] to assess trait anxiety and the Beck Depression Inventory-II (BDI-II [63]).

Finally, participants provided information on: previous CRC screening tests, other cancer screening test(s), family members or close friends with CRC cancer, age, sex, occupation, and education, using an ad-hoc paper-based self-report questionnaire. After completing the study, participants were thanked and debriefed.

#### Data reduction and analysis

A digital trigger detecting R-waves was applied to the ECG signal to obtain interbeat intervals, which were then converted to heart rate (HR). The raw EMG signal was rectified and then smoothed using a moving average filter with a time interval of 500 ms.

Physiological signals were recorded continuously for 30 s immediately prior to narrative onset and throughout the reading. For all the physiological measures, the time course of each response during each narrative reading was analysed by dividing the reading times of each participant into two time intervals. For HR and corrugator EMG, change scores between each epoch and the last 5-s baseline interval were analysed. For SCL, the maximum change (relative to baseline) occurring within each epoch was considered. A log transformation was used to normalize the SCL data.

As far as physiological responses are concerned, since anxiety (both state and trait) and depression are known to affect emotional reactivity, we controlled for their effects by considering STAI-Y1, STAI-Y2 scores and BDI-II scores as covariates. Since trait anxiety and depression often co-occur [64], we calculated multicollinearity statistics. The results confirmed that the correlation between STAI-Y2 scores and BDI-II scores was significant (r = .67, p < .001), nevertheless multicollinearity was reasonably low (tolerance values > .49 and variance inflation factors < 2.03), and therefore both scores were retained as covariates. Physiological measures were analysed using mixed ANCOVA models, with STAI-Y2 scores and BDI-II scores as covariates, with *Narrative Emotion Group* as a between-participants factor, and *Narrative Content* and *Time* (time 1, time 2) as within-participants factors.

In the case of subjective emotional responses, only depression was included in the model as a covariate, because anxiety was concurrently assessed as dependent variable. Subjective emotional responses were analysed with a mixed analysis of covariance (ANCOVA) model, with BDI-II scores as covariate, with *Narrative Emotion Group* (EE vs. NEE) as a between-participants factor, and *Emotion Type* (fear, anger, sadness, disgust, surprise, joy, and anxiety) and *Narrative Content* (control, regret-based, relief-based) as within-participants factors.

Intention to undergo screening was analysed with an ANOVA model, with *Narrative Emotion Group* (EE vs. NEE) as between-participants factor, and *Narrative Content* (control, regret-based, relief-based) as within-participants factor. Additionally, for each type of narrative, a series of regression analyses were performed on intention to undergo screening with subjective emotional measures, HR changes, corrugator EMG changes, and logSCL changes as separate predictors.

The corrected p-values for effects within variables with more than two levels are reported together with the Greenhouse-Geisser epsilon (e) and the uncorrected degrees of freedom. Effect sizes are reported using partial eta-squared (η^2^_p_). Significant main effects and interactions (p < .05) were followed by Tukey HSD post-hoc tests.

### Results

The socio-demographic characteristics of the sample are reported in Table 1. Baseline randomization checks were conducted and showed no differences (S4 Appendix).

Baseline intention to undergo screening was high (*M* = 8.52, *SD* = 1.05). The ANOVA revealed no significant main effects of *Narrative Content* (*p* = .508; relief-based narrative *M* = 8.53, *SD* = .97; regret-based narrative: *M* = 8.48, *SD* = .97; control narrative: *M* = 8.50, *SD* = 1.02), *Narrative Emotion Group* (*p* = .195; EE: *M* = 8.35, *SD* = .96; NEE: *M* = 8.68, *SD* = .96), and no significant interaction (*p* = .869). None of the regression models on intention was significant (all *p*s > .30).

**Fig 2:**
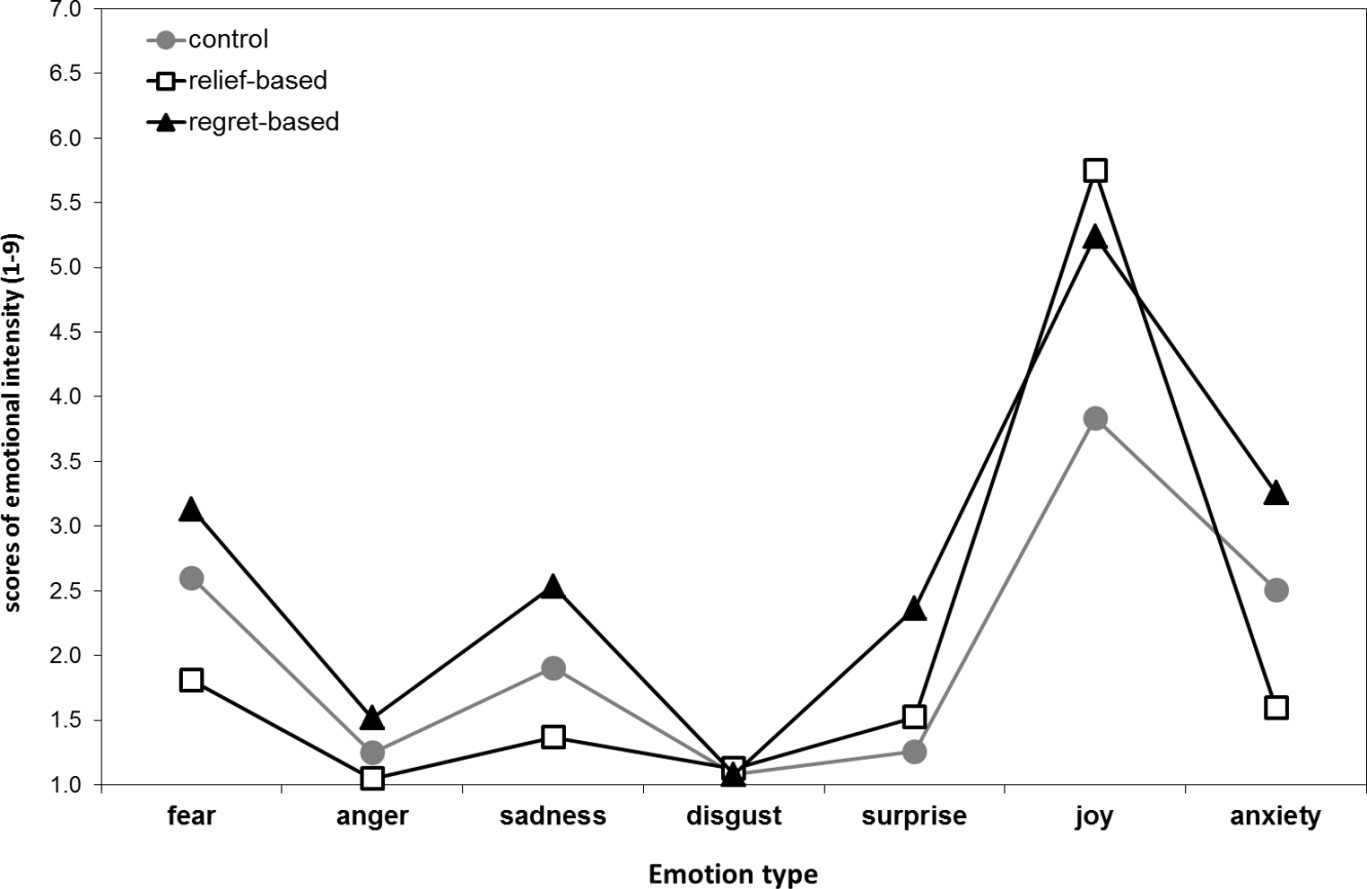
Emotions. Intensity of the emotions experienced after the reading of each narrative as a function of narrative type, independent of the narrative emotion group (with or with no expressed emotions).

As for subjective emotional responses, the ANCOVA highlighted significant main effects of *Narrative Content* (*F*_2, 114_ = 6.31, *p* < .003, ε = .92, η^2^_p_ = .87), and *Emotion Type* (*F*_6, 342_ = 34.84, *p* < .0001, ε = .44, η^2^_p_ = 1.00). Independent of emotion type, the relief-based narrative elicited the highest emotional intensity (all *ps* < .0002), while independent of narrative type, the emotion most intensely felt was joy (all *ps* < .0001), followed by fear, sadness and anxiety. As specified by the significant *Narrative Content* × *Emotion Type* interaction (*F*_12, 684_ = 2.97, *p* < .01, ε = .45, η^2^_p_ = .87), both the relief-based and the regret-based narratives elicited significantly more joy than the control narrative (*p* < .0001). Importantly, the relief-based narrative elicited significantly less anxiety than the control narrative (*p* < .02) and significantly less fear (*p* < .0001), sadness (*p* < .0001), surprise (*p* < .05), and anxiety (*p* < .0001) than the regret-based narrative (Fig 2). No other effects were significant (all *ps* > .23). Furthermore, the post-hoc tests highlighted that the emotion most intensely felt after the reading of the relief-based narrative was joy (all *p*s < .0001), with no other emotion showing a significantly higher intensity; in contrast, for the control and the regret-based narratives, besides joy, fear and anxiety clearly prevailed over anger, disgust, and surprise (all *p*s < .0001).

**Fig 3:**
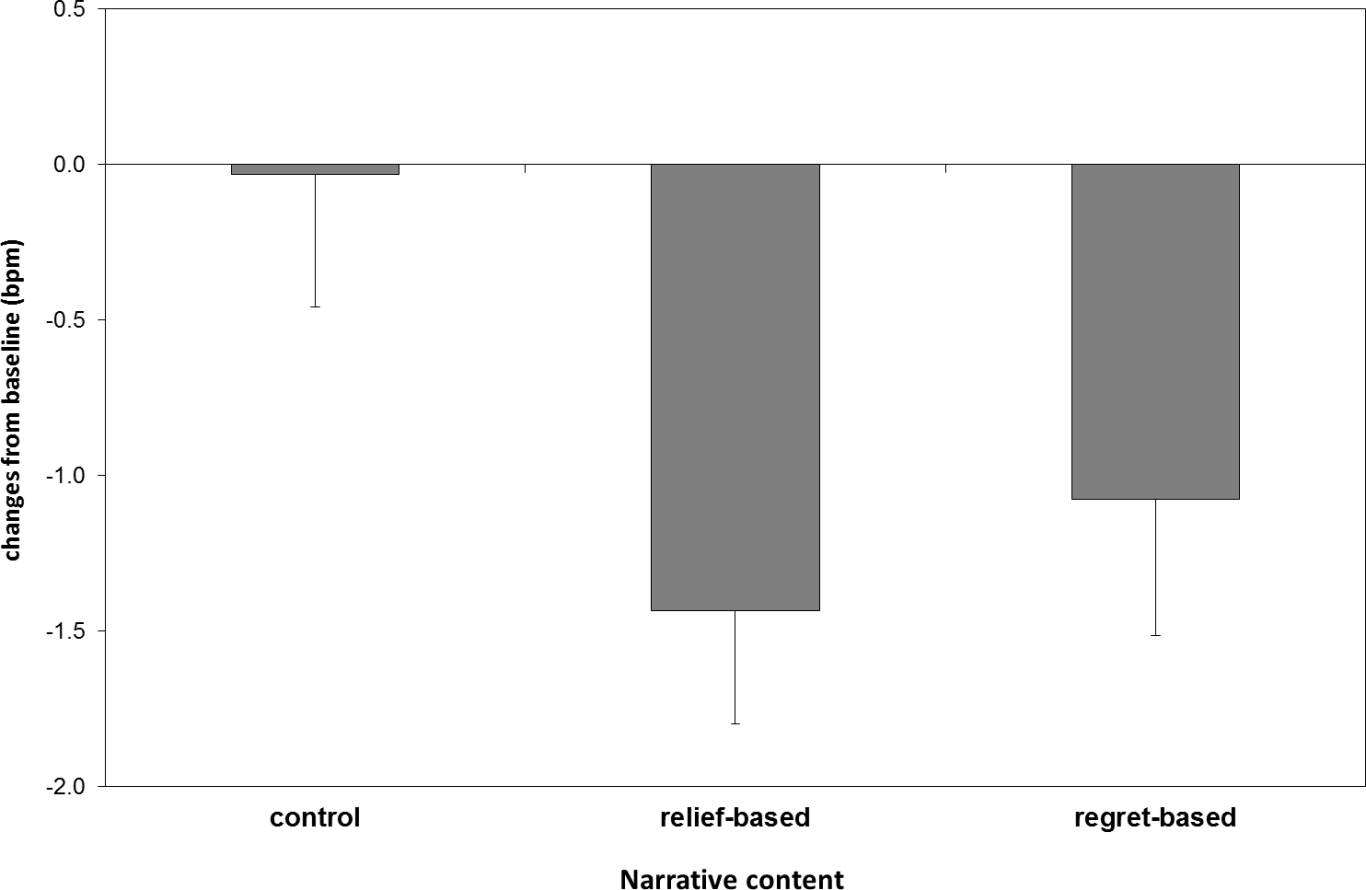
Heart rate. HR decreases during narrative reading as a function of narrative content.

The ANCOVA on HR responses revealed a significant main effect of *Narrative Content* (*F*_2, 108_ = 3.59, *p* = .033, ε = .95, η^2^_p_ = .06) (Fig 3). No other effects were significant (all *ps* > .17). Post-hoc tests showed that the relief-based narrative elicited a greater HR decrease than the control narrative (*p* = .047). No significant difference emerged between the regret-based and the control narratives (*p* = .20) or between the regret-based and the relief-based narratives (*p* = .78).

**Fig 4:**
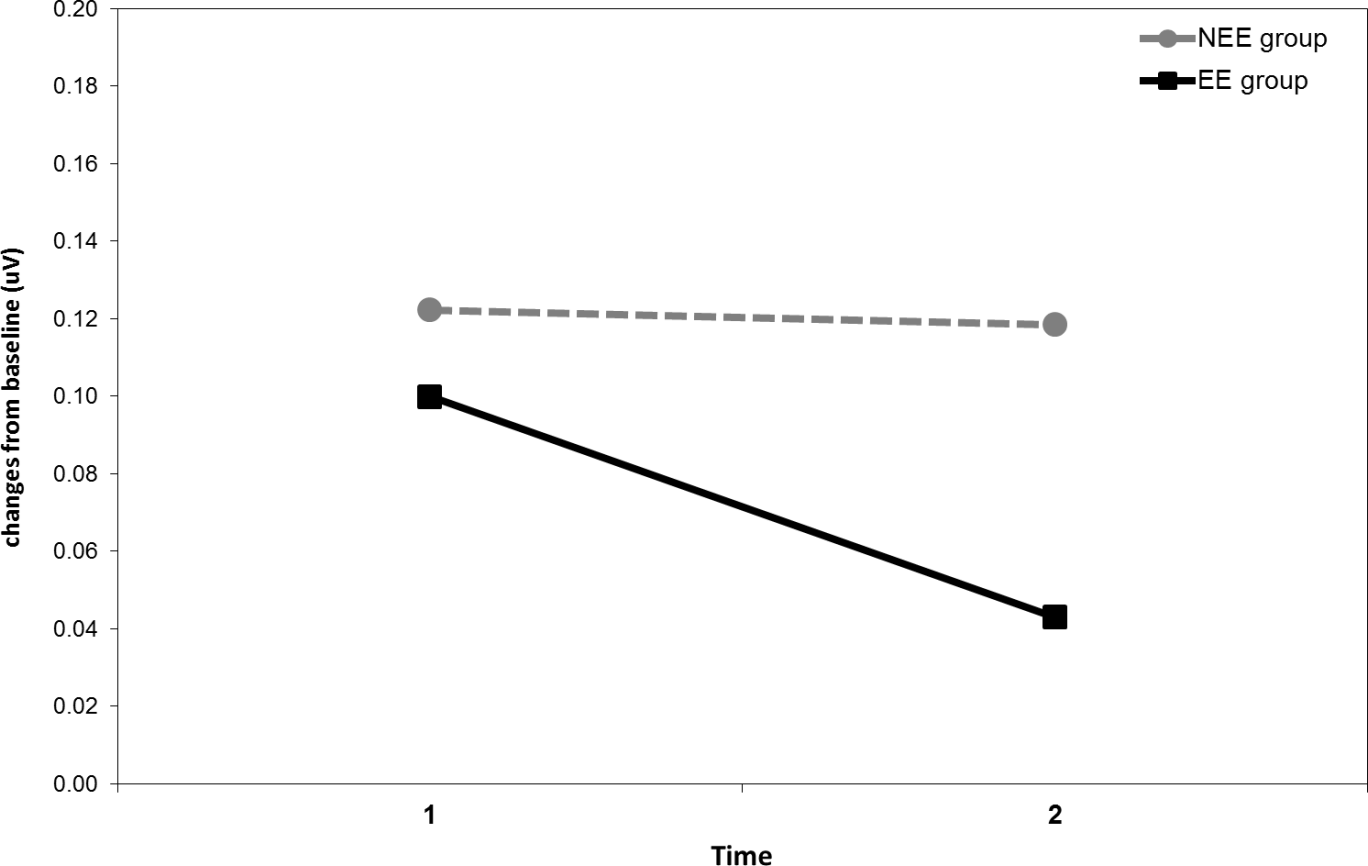
Corrugator EMG. Corrugator EMG activity during narrative reading across time as a function of the narrative emotion group (NEE = with no expressed emotions; EE = with expressed emotions).

For EMG a significant main effect of *Time* was found (*F*_1, 55_ = 11.89, *p* = .001, η^2^_p_ = .92), showing an overall decrease in corrugator activity from time 1 to time 2, indicating decreased negative affect. However, as specified by the significant *Narrative Emotion Group* × *Time* interaction (*F*_1, 55_ = 4.09, *p* < .048, η^2^_p_ = .51), the EE group showed a significant decrease in corrugator EMG activity from time 1 to time 2 (*p* = .046), whereas the NEE group did not show any change between the two time intervals (*p* = .99) (Fig 4). No other effects were significant (all *ps* > .33).

The analysis of logSCL revealed a significant main effect of the *Narrative Emotion Group* (*F*_1, 55_ = 4.03, *p* = .049, η^2^_p_ = .51), with the EE narratives inducing lower overall activation than the NEE narratives (*Ms* = .043 and .025 uS, respectively). No other effects were significant (all *ps* > .19).

### Discussion

No differences in intention to undergo screening emerged as a function of the different narrative contents. However, baseline intention to undergo screening measured before reading any narrative was very high, suggesting that the lack of significant effects might be attributable to a ceiling effect. A possible explanation is that participants might have been highly motivated to participate in this study because of their particular interest in health behaviours. However, we cannot exclude that such ceiling effect was related to social desirability.

Nonetheless, the emotional response patterns consciously elicited by the three narratives were clearly different, and the physiological changes observed during the reading provided relevant information on the cognitive and affective processes differentially engaged by the narratives. In the General Discussion, we discuss the main findings and how they relate to the results obtained in Study 1.

## General discussion

Two studies investigated the effects of narratives with different emotional contents in promoting informed uptake of FIT as a screening test for CRC. Study 1 confirmed that narratives are promising tools to promote CRC screening, as previously suggested [13–16]. However, not all narratives were equally effective in promoting CRC screening, supporting the differential effect of different emotional contents on persuasion [33].

While the existing literature suggested both anticipated regret and relief as good candidates to motivate CRC screening, our findings provide evidence for the superiority of relief. Indeed, the relief-based narrative yielded the highest intention to undergo testing, whereas the anticipated regret-based narrative did not differ from the no-narrative condition or from the control narrative in terms of intention. It should be noted that the story used in our anticipated regret-based narrative was relatively positive, as cancer was found early and it was treated successfully. More dramatic stories might be effective [25], but, as suggested by decades of studies on fear appeal messages, excessive threat may induce defensive reactions, including denying or questioning the severity of the threat or the susceptibility to it [36]. Indeed, there is some evidence that having people imagine not to do the test and later find out to have CRC does not increase intentions to be screened [38]. Moreover, there is qualitative evidence that hearing about others’ bad experience can discourage people from screening [65–66]. Our findings contribute to shed light on the mixed evidence on the effectiveness of narratives in promoting CRC screening.

Additionally, extending previous studies, we aimed to systematically test the effects of different aspects of the narrative. By including a control narrative condition, where the character reported having undergone the FIT test and awaiting for the test result, we aimed to disentangle the effects of simply modelling the behaviour (i.e., “I did the test”), from those provided by the additional information included in the other two narratives. However, it has to be noted that awaiting test results is not a neutral situation, as shown by the emotional responses obtained in Study 2 (intermediate levels of anxiety, fear, and sadness relative to the other two narratives). In order to improve the experimental control over the narrative content, in future studies the control narrative could encompass the story of someone who did the test and received the results, but does not disclose them in the narrative [23].

Contrary to previous concerns regarding reduced attention to factual information [59–60], the results of Study 1 showed that adding a narrative to the standard material did not bias the critical information to be conveyed: knowledge was not decreased, risk perception was not altered, and the proportion of informed choices was comparable, relative to the standard information material. A similar finding was previously reported—for knowledge only–when comparing patient decision aids with or without narrative(s) that included similar information content [17]. Therefore, it seems critical that narrative information is used as supplementary information rather than as an alternative format. This finding has important implications for screening programme communications.

As for Study 2, to the best of our knowledge, this is the first study using psychophysiological methods to assess both explicit and implicit indicators of the emotional processes engaged by patient narratives in the context of CRC screening. Together with self-reported emotions, the time course of somatic and autonomic measures was recorded during the reading of the relief-based, anticipated regret-based, and control (i.e., test-uptake only) narratives. In addition, the effects of the explicit description of the emotions felt by the narrative character were examined in a between-participants design (expressed emotions, EE, vs. non expressed emotions, NEE).

Despite no differences in intention to undergo screening emerged as a function of the different narrative contents, probably due to a ceiling effect, the subjective emotional responses experienced after the reading of the three narratives were clearly different. The relief-based narrative elicited significantly less intense negative emotions (i.e., fear, sadness, anxiety) and less intense surprise than the anticipated regret-based narrative, and a more intense positive emotional state (i.e., joy) than the control narrative. These emotions might be elicited by different aspects of the narrative stories. For example, while joy reported for the relief-based narrative is likely to be related to the test result, for the regret-based narrative it is likely to refer to the fact that the ending of the story is positive, i.e., a cancer was found but it was early-stage and it was successfully treated. Indeed, while joy is the emotion prevailing for the relief-based narrative, the regret-based narrative induced high levels of fear and anxiety, besides joy. It is worth noting that while the feeling of joy might seem an unexpected finding, both the relief and the anticipated regret narratives refer to a possible negative outcome that was avoided. Moreover, all of our narratives describe someone who has already decided to undergo screening. Therefore, having decided to perform the screening behaviour might contribute to this positive emotional response. Indeed, even in the control narrative the level of reported joy is not trivial. As previously advocated, stories are complex interventions [18–19]; even relatively short written narratives, like those used in our studies, are likely to be complex stimuli, eliciting several conscious and unconscious emotional responses. Although it is difficult to investigate the effects of different emotions on persuasion [67], the results of Study 2, together with the effects on intention found in Study 1, suggest that relief-based stories may lower negative emotions and be more persuasive, in the context of FIT for CRC screening.

It is worth noting that, while different pattern of subjective emotional responses were found for the three narratives, the intensity of the emotions reported was similar regardless of whether the narrative character explicitly expressed her emotions or not. This result suggests that adding a description of the emotions felt by the narrative character at the end of the story does not alter the *conscious* emotional experience of readers.

Relevant information on the nature of the motivational processes evoked during narrative reading was provided by the analysis of the physiological measures. Interestingly, the different physiological variables provided different and complementary information.

Heart rate (HR) changes were particularly sensitive to the narrative content, in that larger HR decreases were specifically observed for the relief-based than the control narrative, independent of whether emotions were expressed by the narrative character or not. This result, taken together with the overall increase in skin conductance, indicates a state of enhanced attention and orienting [40] during the reading of the relief-based narrative. In contrast, this was not the case for the anticipated regret-based narrative. Therefore, our data suggest that a relief-based narrative not only is able to reduce the intensity of the negative emotions experienced, as indicated by the subjective emotional measures, but it also has more attention-grabbing power, possibly helping to maintain the focus of attention on screening-related information.

On the other hand, skin conductance (SCL) and corrugator electromyographic (EMG) changes were particularly sensitive to the presence of emotions explicitly expressed by the narrative character, which only affected *unconscious* measures. Specifically, participants in the EE condition showed lower SCL activity during the reading of the narratives, indicating lower physiological arousal, and a decrease in corrugator EMG activity from the first to the second time interval, indicating decreased negative affect during reading. Indeed, greater activity of the corrugator (frown) muscle is associated with processing of unpleasant events [39]. It is worth noting that in the EE condition the text depicting the emotions expressed by the narrative character was placed at the end of each narrative, thus corresponding exactly to the second time interval of reading. Taken together, these data reflect a psychophysiological response pattern indicative of reduced negative emotional activation. On this basis, as a further application of our results, we suggest that including in the narrative the description of emotions explicitly expressed by the character might lower the impact of negative emotions in the reader and induce a broad disposition to respond with lower defensive activation, thus facilitating adhesion to the screening program. However, as our data on intention to undergo screening were unable to show any significant increase, probably due to a ceiling effect linked to the high motivation of participants and to the setting of the study, future studies are needed in order to ascertain whether including the emotions of the narrative character contribute to the persuasiveness of the narrative, as previously suggested [19]. Additionally, future studies might benefit from using alternative anchor points on the intention to screen measure to reduce the effect of high motivation and social desirability.

The present studies have some limitations. First, it should be noted that the narratives did not differ only in terms of emotional content but also in terms of the test result: relief was associated with a negative test result whereas anticipated regret was associated with a positive test result and an early stage cancer diagnosis. From a methodological perspective, ideally we should have manipulated both the test result and the emotional content. Unfortunately, it is extremely improbable and unrealistic to associate anticipated regret with a negative test result and relief with a positive test result per se, although relief could be associated with the fact that cancer was identified early, and it was successfully removed.

Second, although there is evidence that intention to engage in a behaviour is highly predictive of the engagement with the behaviour itself [68], there is also a gap or discrepancy between intention and behaviour [69–70], therefore our findings should be replicated with real CRC invitation letters. Still, caution should be paid when designing the study in real settings, as additional factors may play a role. For instance, in a large randomised study on actual uptake of screening, McGregor et al. [21] failed to replicate their previous findings established with intention measures [20]. However, due to organisational reasons, their narratives were not sent together with the FOBT kit and its instructions, but with a pre-invitation letter. As suggested by the authors, “had the leaflet accompanied the test kit, individual may have been more likely to attend to it and it may have additionally acted as a point-of-choice prompt and subsequently had a stronger impact on actual test completion.” (p7) [21].

Another limitation of the present research is the possibility of self-selection, i.e., as previously noted, participants who agreed to participate may have had more positive attitudes towards screening than those declining participation, especially for Study 2, requiring greater time and dedication. Nevertheless, this attitude, as well as social desirability, is likely to affect conscious responses, especially intention, to a greater extent, but is less likely to affect physiological changes during the reading.

Finally, different narratives with different emotional contents could be examined in future studies, including disgust, which has been found to be a barrier to screening, although only for a minority of participants [71–72]. Dillard and colleagues [23] were able to tailor narratives to specific barriers held by their participants, including disgust. While this approach would be very difficult to use when sending mass invitation letters in public health campaigns, narratives reporting disgust might be useful to follow-up with non-screeners.

We view our results as providing useful pieces of a puzzle in which several psycho-physiological aspects must be considered in order to better understand the promising nature of narratives in encouraging CRC screening.

## Supporting information

S1 Dataset Excel file with data from Studies 1 and 2. (XLSX)

S1 Appendix Invitation letter and information leaflet. Invitation letter and information leaflet adapted from (translated from Italian).
(DOCX)

S2 Appendix Narratives. Narratives used in Study 2 (translated from Italian). The text reported here in Italics was shown only in the condition with expressed emotions (EE).
(DOCX)

S3 Appendix Baseline randomization checks for Study 1. (DOCX)

S4 Appendix Baseline randomization checks for Study 2. (DOCX)
