# Supplementary material for: Assessing emotions conveyed and elicited by patient narratives and their impact on intention to participate in colorectal cancer screening: A psychophysiological investigation
Source: PLoS One. 2018 Jun 28;13(6):e0199882. doi: 10.1371/journal.pone.0199882 (PMC6023155; doi:10.1371/journal.pone.0199882)
Supplement: S1 Appendix — Invitation letter and information leaflet adapted from (translated from Italian). (DOCX) [file pone.0199882.s002.docx]

**S1 Appendix. Invitation letter and information leaflet.** Invitation letter and information leaflet adapted from (translated from Italian).
