# Supplementary material for: Assessing emotions conveyed and elicited by patient narratives and their impact on intention to participate in colorectal cancer screening: A psychophysiological investigation
Source: PLoS One. 2018 Jun 28;13(6):e0199882. doi: 10.1371/journal.pone.0199882 (PMC6023155; doi:10.1371/journal.pone.0199882)

**S2 Appendix. Narratives.** Narratives used in Study 2 (translated from Italian). The text reported here in Italics was shown only in the condition with expressed emotions (EE).

**Reassurance-based narrative**

**
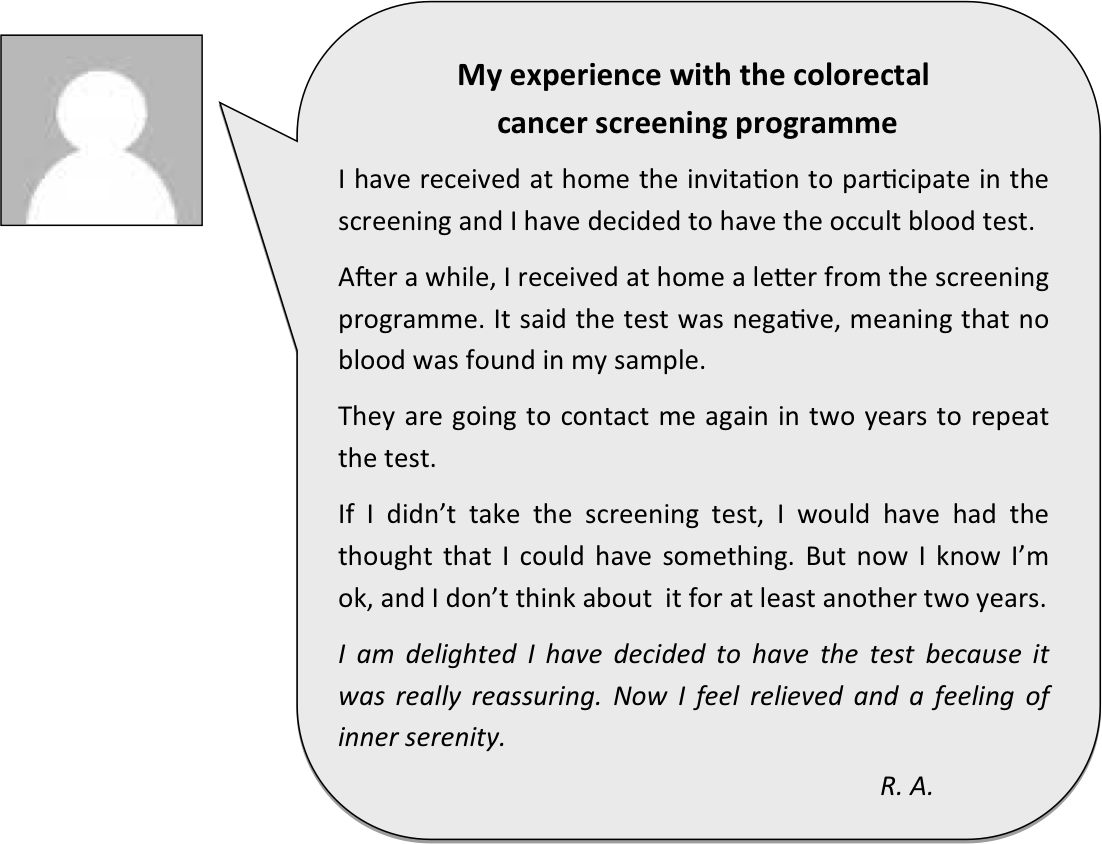
**

**Regret-based narrative**

**
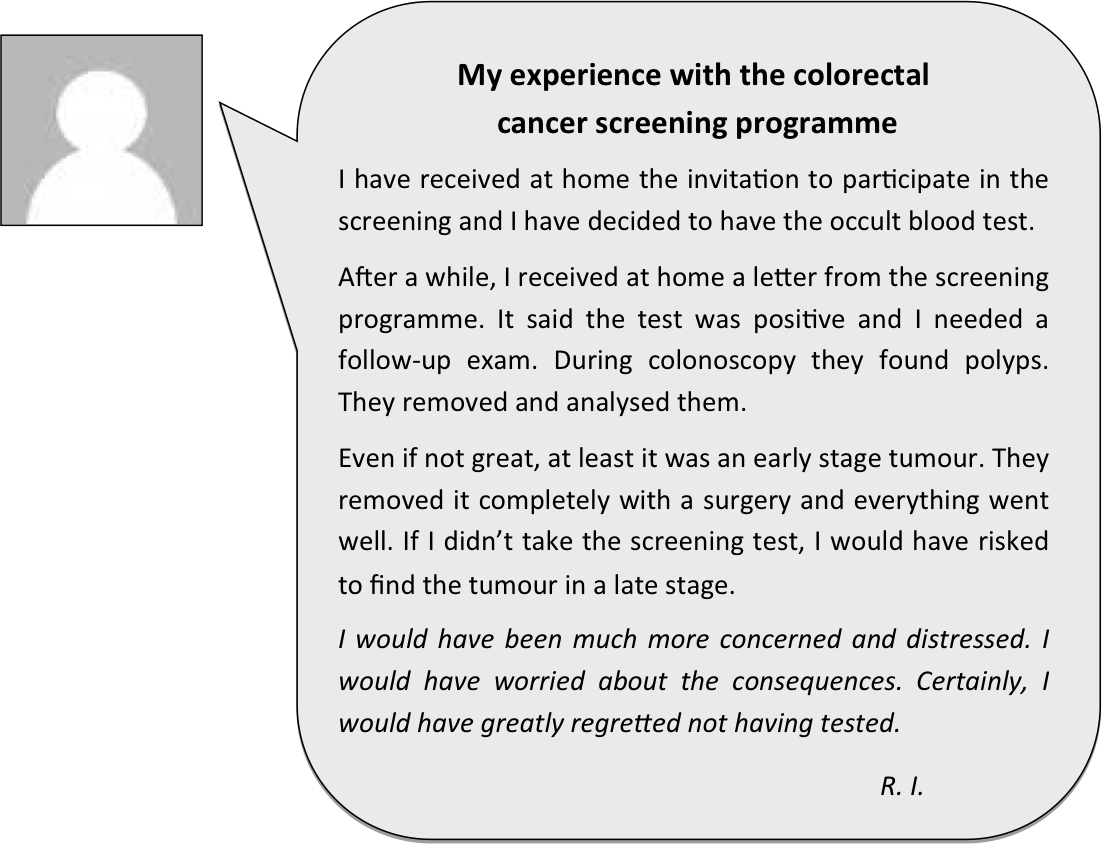
**

**Control narrative**


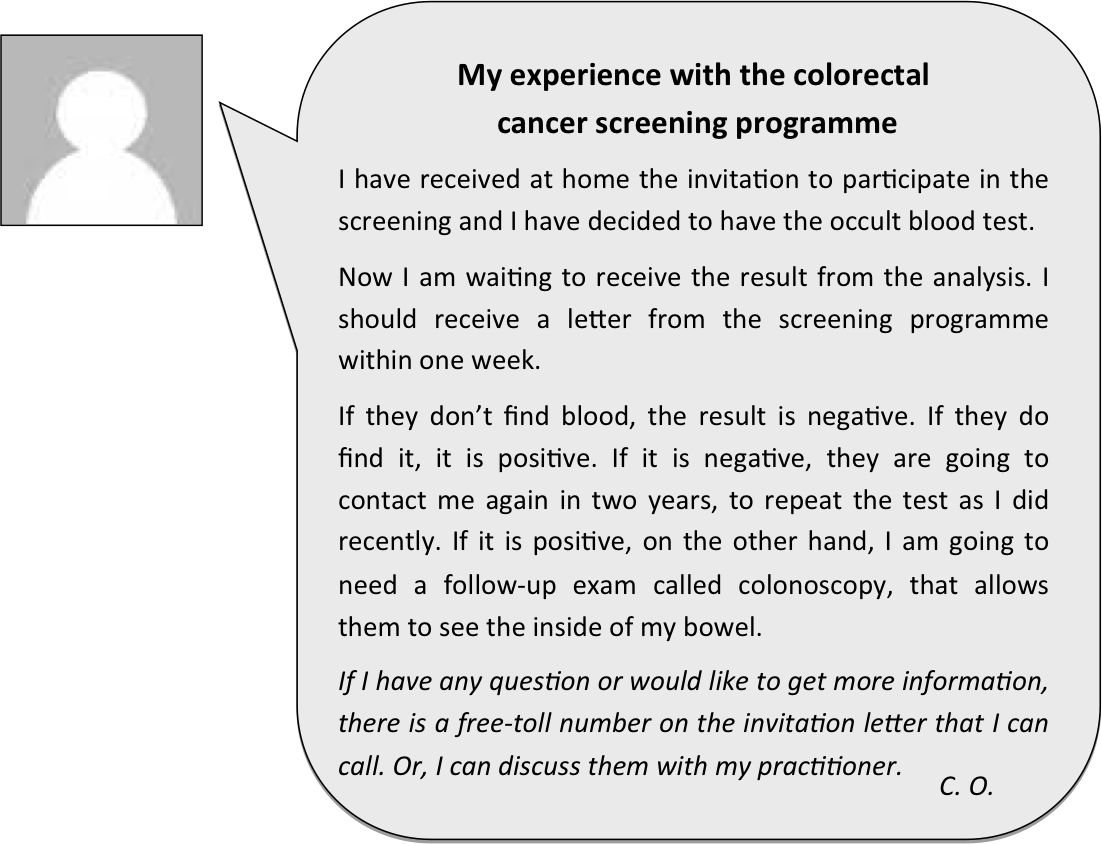


**
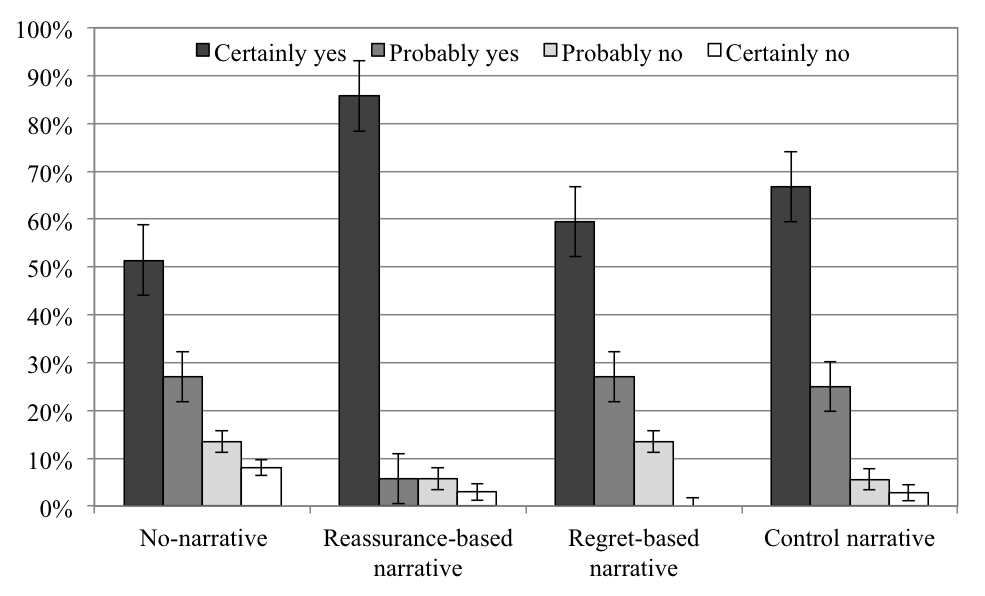
**


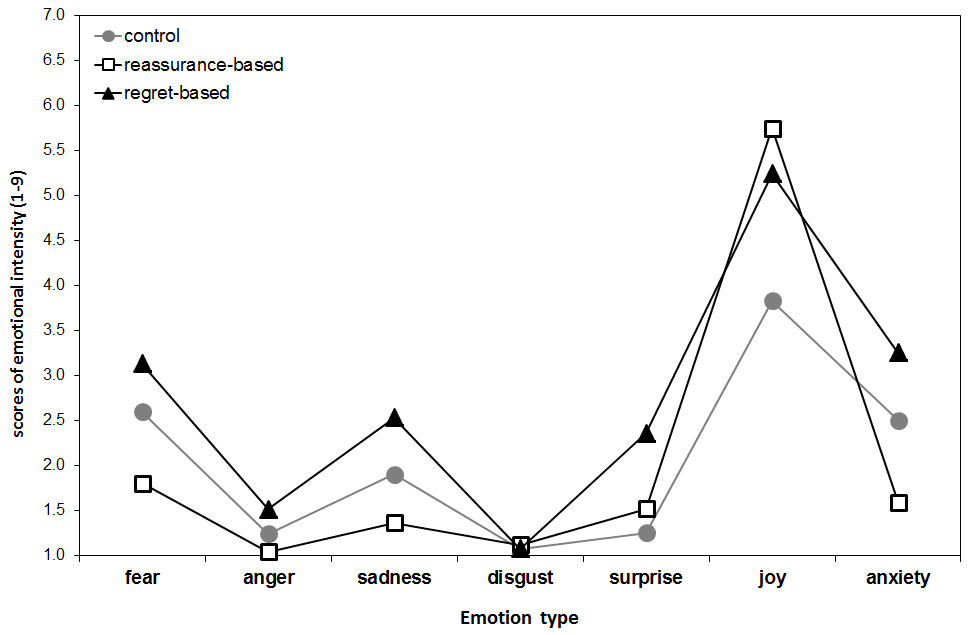


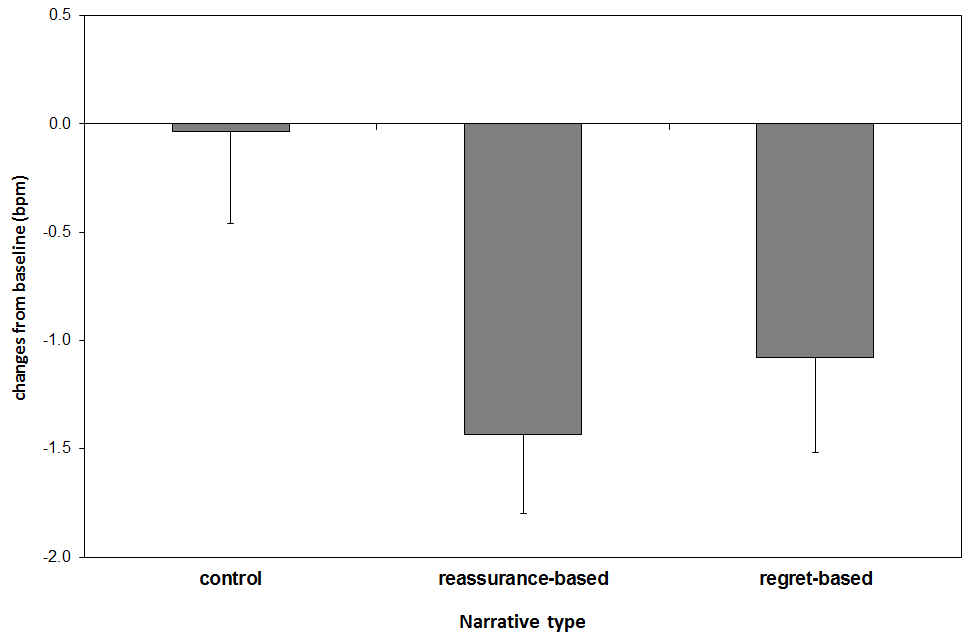


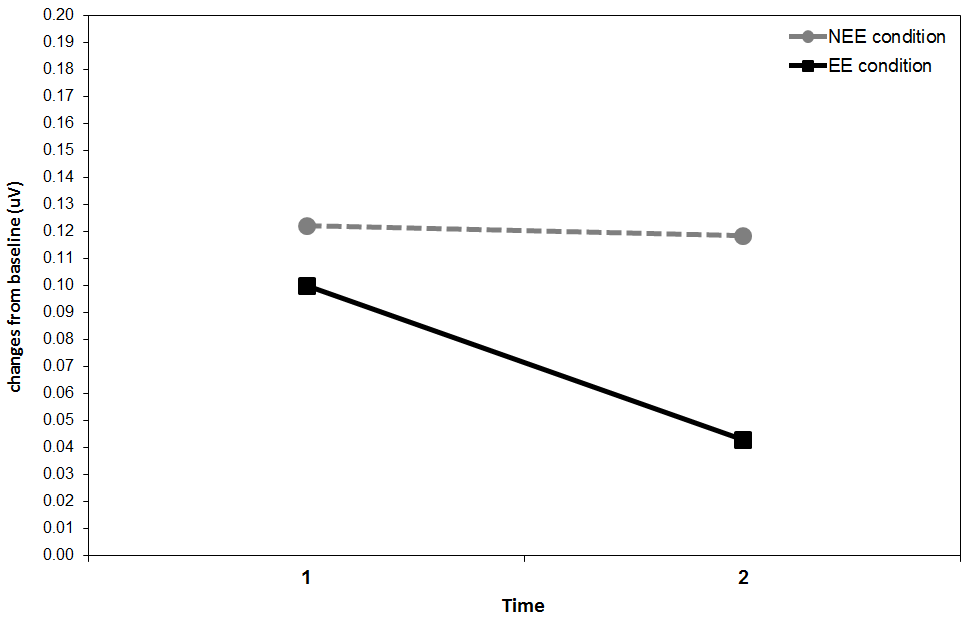

Supplement: S2 Appendix — Narratives used in Study 2 (translated from Italian). The text reported here in Italics was shown only in the condition with expressed emotions (EE). (DOCX) [file pone.0199882.s003.docx]
