# Supplementary material for: Assessing emotions conveyed and elicited by patient narratives and their impact on intention to participate in colorectal cancer screening: A psychophysiological investigation
Source: PLoS One. 2018 Jun 28;13(6):e0199882. doi: 10.1371/journal.pone.0199882 (PMC6023155; doi:10.1371/journal.pone.0199882)
Supplement: S3 Appendix — (DOCX) [file pone.0199882.s004.docx]

**S3 Appendix. Baseline randomization checks for Study 1.**

|  | No narrative | Control narrative | Reassurance narrative | Anticipated regret narrative | Test of difference |
| --- | --- | --- | --- | --- | --- |
| Age [Mean (SD)] | 54.16  (4.953) | 54.06  (6.585) | 54.97  (4.712) | 54.11  (5.216) | F (141) = .227,  p = .878 |
| Sex |  |  |  |  | χ^2^ (3) = .238,  p = .971 |
| F | 19 (51.4%) | 19 (52.8%) | 17 (48.6%) | 20 (54.1%) |  |
| M | 18 (48.6%) | 17 (47.2%) | 18 (51.4%) | 17 (45.9%) |  |
| Education |  |  |  |  | χ^2^ (9) = 10.480,  p = .313 |
| middle school | 6 (17.1%) | 14 (38.9%) | 14 (40.0%) | 8 (22.9%) |  |
| vocational school | 9 (25.7%) | 5 (13.9%) | 6 (17.1%) | 4 (11.4%) |  |
| high school | 13 (37.1%) | 13 (36.1%) | 9 (25.7%) | 17 (48.6%) |  |
| university degree and other | 7 (20.0%) | 4 (11.1%) | 6 (17.1%) | 6 (17.1%) |  |
| Occupation |  |  |  |  | χ^2^ (12) = 14.670,  p = .260 |
| office worker | 13 (35.1%) | 13 (36.1%) | 19 (54.3%) | 15 (40.5%) |  |
| professionals | 12 (32.4%) | 4 (11.1%) | 3 (8.6%) | 5 (13.5%) |  |
| retired | 5 (13.5%) | 7 (19.4%) | 5 (14.3%) | 6 (16.2%) |  |
| housewives | 3 (8.1%) | 8 (22.2%) | 6 (17.1%) | 5 (13.5%) |  |
| other | 4 (10.8%) | 4 (11.1%) | 2 (5.7%) | 6 (16.2%) |  |
