# Supplementary material for: Assessing emotions conveyed and elicited by patient narratives and their impact on intention to participate in colorectal cancer screening: A psychophysiological investigation
Source: PLoS One. 2018 Jun 28;13(6):e0199882. doi: 10.1371/journal.pone.0199882 (PMC6023155; doi:10.1371/journal.pone.0199882)
Supplement: S4 Appendix — (DOCX) [file pone.0199882.s005.docx]

**S4 Appendix. Baseline randomization checks for Study 2.**

|  | NEE | EE | Test of difference |
| --- | --- | --- | --- |
| Age [Mean (SD)] | 47.68 (1.744) | 47.69 (1.575) | t (58) = -.021,  p = .983 |
| Sex | F: 15 (53.6%)  M: 13 (46.4%) | F: 17 (53.3%)  M: 15 (46.9%) | χ2 (1) = .001,  p = .972 |
| Education |  |  | χ2 (3) = 1.443,  p = .695 |
| middle school and  vocational school | 4 (14.3%) | 5 (15.6%) |  |
| high school | 11 (39.3%) | 16 (50.0%) |  |
| university degree | 9 (32.1%) | 9 (28.1%) |  |
| other | 4 (14.3%) | 2 (6.2%) |  |
| Occupation |  |  | χ2 (2) = 1.855,  p = .396 |
| office worker | 17 (60.7%) | 21 (65.6%) |  |
| professionals | 5 (17.9%) | 8 (25.0%) |  |
| housewives and other | 6 (21.4%) | 3 (9.4%) |  |
